# An effective case of pulsatile flow for cerebral malperfusion of stanford type A aortic dissection

**DOI:** 10.1051/ject/2025011

**Published:** 2025-09-15

**Authors:** Tomohisa Takeichi, Yoshihisa Morimoto, Akitoshi Yamada, Takanori Tanaka, Kunihiro Fujiwara, Masanobu Sato, Ryo Toma, Kiyoto Mitsui, Takumi Sugita, Kunio Gan

**Affiliations:** 1 Department of Clinical Engineering, Kitaharima Medical Center 926-250, Ichiba-cho, Ono-shi Hyogo 675-1392 Japan; 2 Department of Cardiovascular Surgery, Kitaharima Medical Center 926-250, Ichiba-cho, Ono-shi Hyogo 675-1392 Japan

**Keywords:** Malperfusion, Pulsatile flow, Aortic dissection, Near-infrared spectroscopic oximetry (NIRO)

## Abstract

The surgical management of preoperative malperfusion poses considerable challenges, particularly in cases of acute type A aortic dissection (TAAD). Herein, we describe the case of a 78-year-old female patient presenting with TAAD complicated by malperfusion of the left lower extremity and an entry tear localized to the ascending aorta. During the initiation of cardiopulmonary bypass (CPB), a pronounced bilateral discrepancy in radial mean arterial blood pressure (mABP) was identified, alongside a significant reduction in cerebral tissue oxygenation index (TOI) and the oxyhemoglobin change rate (ΔHbO_2_). To mitigate the malperfusion, pulsatile flow (PF) was utilized during CPB. This report elucidates the meticulous application of PF during CPB in the management of this complex malperfusion scenario, culminating in a favorable postoperative outcome.

## Introduction

Acute type A aortic dissection (TAAD) outcomes are profoundly influenced by complex malperfusion syndromes, which substantially elevate mortality rates [1]. Lower-limb malperfusion is reported in approximately 20–30% of TAAD cases [2–6]. Although femoral artery cannulation is commonly utilized in the management of TAAD, it is associated with several inherent limitations. Alternatively, strategies incorporating axillary and femoral artery cannulation or employing ascending aorta (Asc Ao) cannulation have demonstrated improved postoperative clinical outcomes [7–9].

In cases complicated by malperfusion, the selection of an appropriate perfusion strategy is paramount. In the presented case, a decision was made to utilize a combination of right femoral artery (FA) and Asc Ao cannulation. However, due to severe narrowing of the true lumen and pronounced mobility of the intimal flap, aortic cannulation was technically challenging. As a result, cardiopulmonary bypass (CPB) was initiated via right FA cannulation. Following the establishment of CPB, a significant bilateral discrepancy in radial mean arterial blood pressure (mABP) was observed. Concurrently, cerebra tissue oxygenation index (TOI) and ΔHbO_2_ demonstrated notable declines. To mitigate malperfusion, the perfusion mode was transitioned from non-pulsatile flow (NPF) to pulsatile flow (PF).

This case report was approved by the Institutional Review Board at Kitaharima medical center (IRB 06-34) with the waiver of informed consent.

## Case report

**Figure 1:**
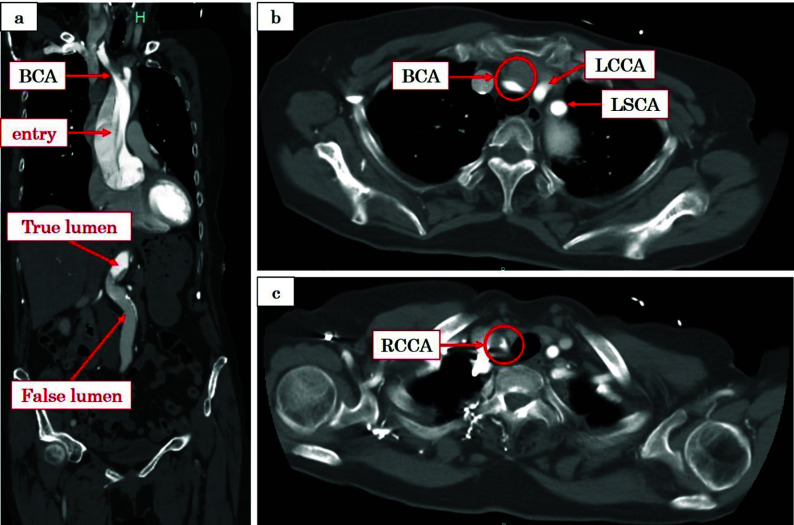
(a) Aortic contrast-enhanced CT scan indicates a DAA with malperfusion of the left lower limb and an entry tear in the ascending aorta. (b) LCCA and LSCA show no abnormalities. (b, c) The true lumen from the BCA to the RCCA is narrowed by the false lumen. CT: Computed tomography; DAA: Dissecting aortic aneurysm; LCCA: Left common carotid artery; LSCA: Left subclavian artery; BCA: Brachiocephalic artery; RCCA: Right common carotid artery.

The patient, a 78-year-old woman (height: 155.3 cm; weight: 49.7 kg), was transported to our hospital following the acute onset of back pain and impaired mobility in the left lower limb. Her medical history was significant for annuloaortic ectasia, previously monitored at our institution. Upon examination in the emergency department, the patient was alert and oriented. Contrast-enhanced computed tomography (CT) revealed a type A aortic dissection (TAAD) with malperfusion of the left lower limb and an entry tear in the ascending aorta (Fig. 1a). The left common carotid artery (LCCA) and left subclavian artery (LSCA) were unremarkable (Fig. 1b). The true lumen was compressed by the false lumen, extending from the brachiocephalic artery (BCA) to the right common carotid artery (RCCA) including the right axillary artery (Figs. 1a–1c). Emergency surgery, including a Bentall procedure and hemiarch replacement, was indicated.

**Figure 2:**
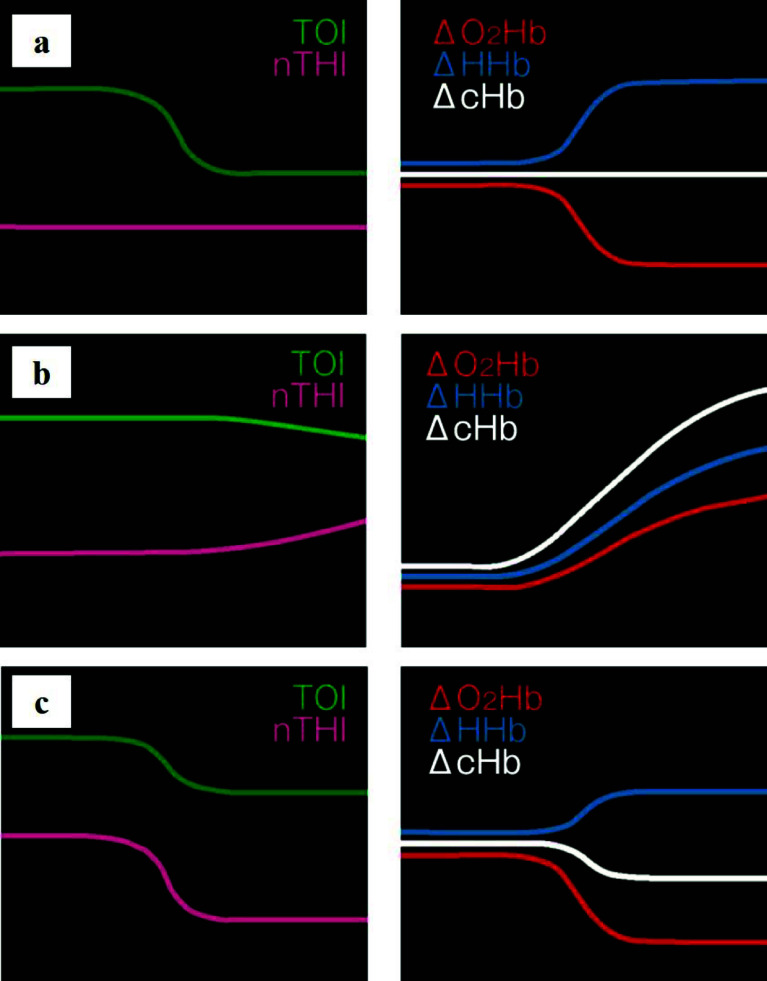
Mechanism of NIRO-200NX. (a) When oxygenation is insufficient, there is no observable change in the inflow of hemoglobin to the brain (nTHI, ΔcHb); however, ΔO_2_Hb decreases, while ΔHHb increases conversely. (b) In cases of congestion, the total hemoglobin volume (nTHI, ΔcHb) increases alongside the rise in ΔHHb in the brain. (c) In ischemic conditions, the inflow of hemoglobin to the brain (nTHI, ΔcHb) decreases.

**Figure 3:**
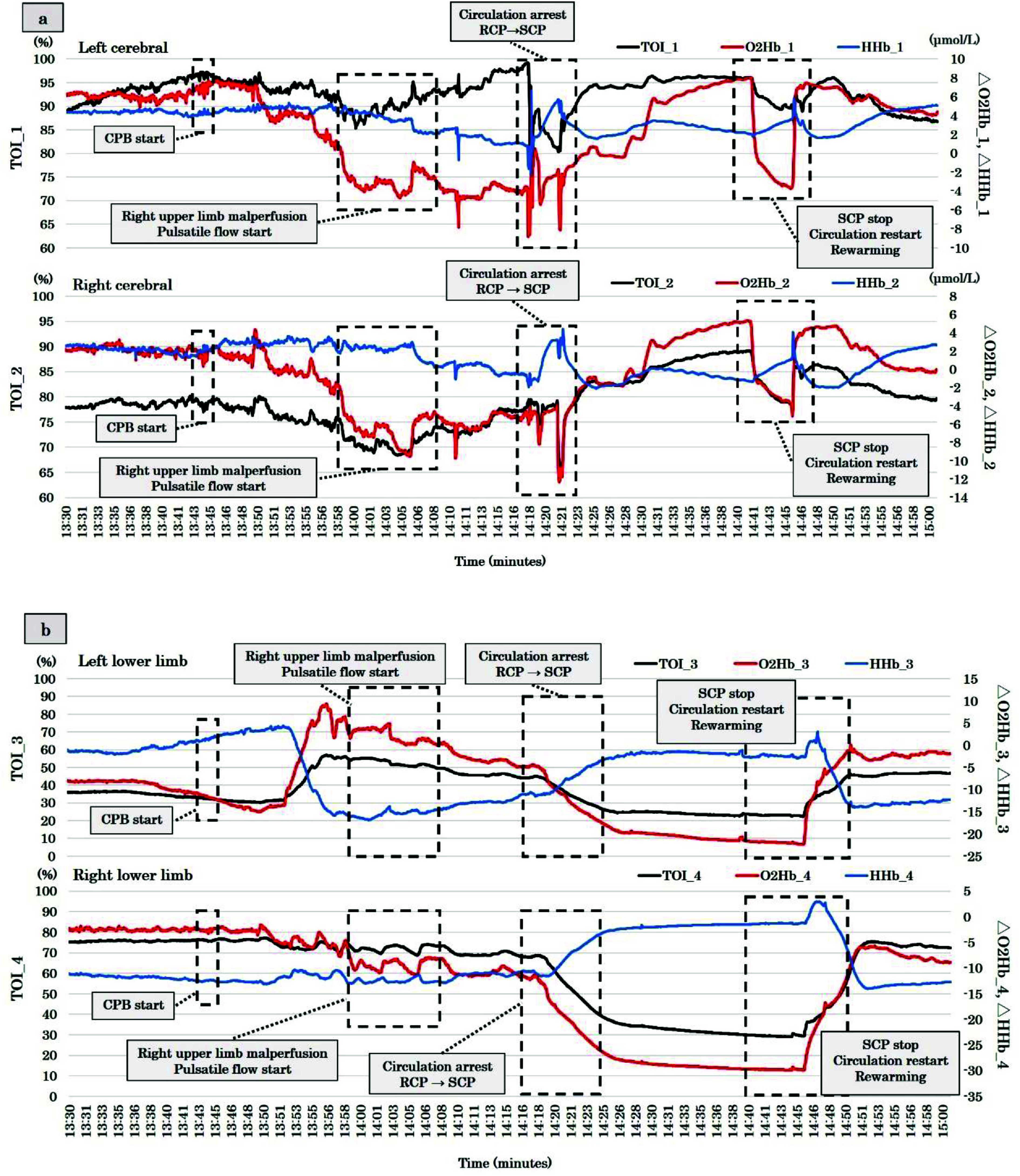
(a, b) The transition of TOI, ΔO_2_Hb, and ΔHHb between the cerebral and lower limb. TOI: Tissue oxygenation index; ΔO_2_Hb: Oxyhemoglobin change rate; ΔHHb: Deoxyhemoglobin change rate.

Following induction of general anesthesia, bilateral radial arterial and central venous catheterizations were performed for three-site pressure monitoring. Intraoperative neurovascular and lower-limb perfusion was assessed using near-infrared spectroscopic oximetry (NIRO 200, Hamamatsu Photonics, Hamamatsu, Japan). The mechanism of NIRO-200NX is shown in Figures 2a–2c. Despite the significant narrowing of the true lumen at the BCA, bilateral radial pressures were maintained between 106/43 and 113/44 mmHg. Cerebral TOI values remained stable at 88–90%, with no interhemispheric differences. However, TOI in the right lower limb was 75%, while it was markedly reduced to 35% in the left lower limb, reflecting malperfusion (Figs. 3a and 3b). Arterial lactate levels were 1.5 mmol/L, indicating no evidence of metabolic derangement.

**Figure 4:**
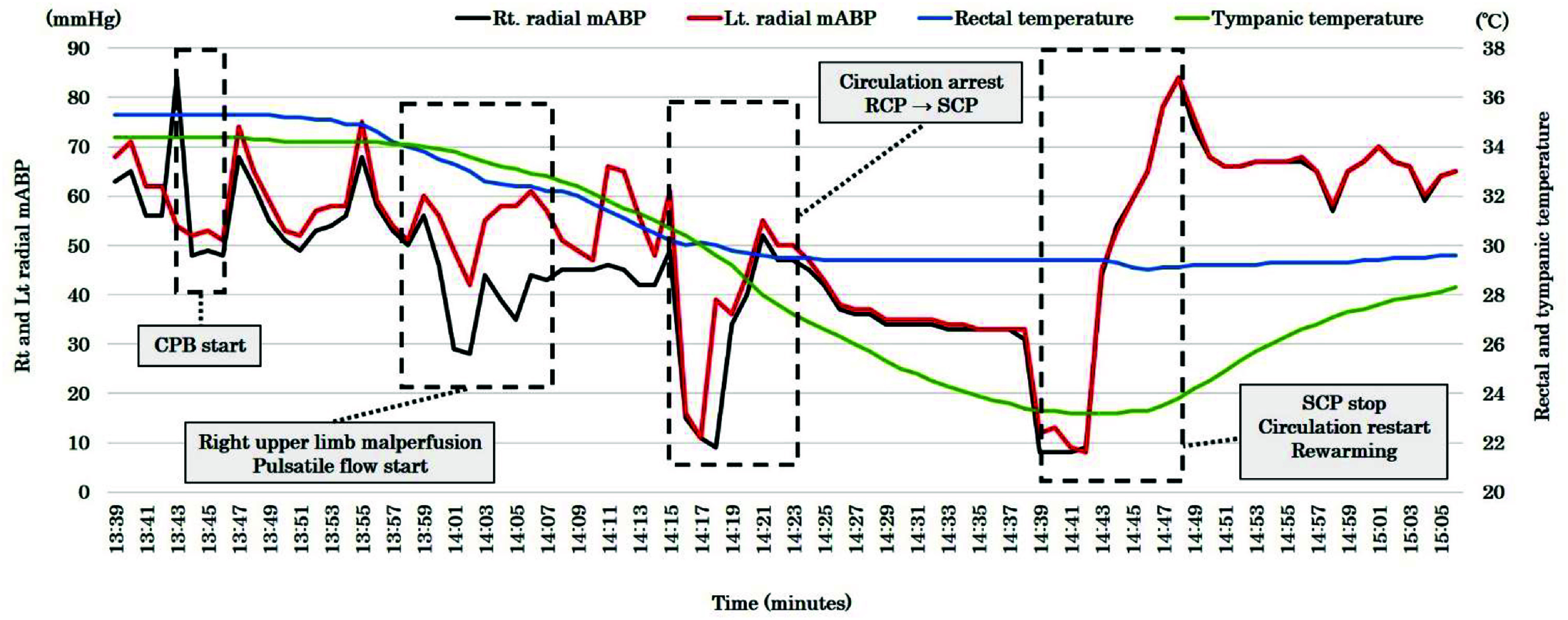
The transition of radial mABP, rectal temperature, and tympanic temperature. mABP: Mean arterial blood pressure.

**Figure 5:**
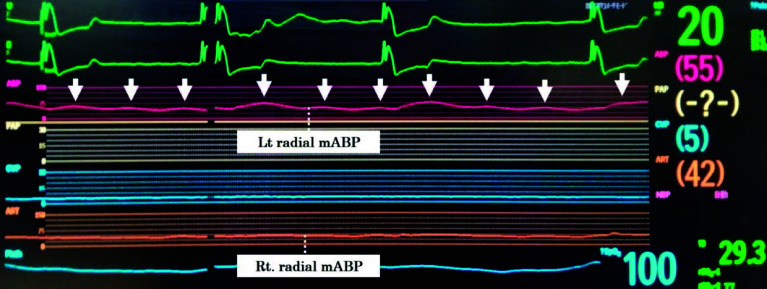
The radial mABP wave after setting PF flow. mABP: Mean arterial blood pressure.

Given the severity of malperfusion, we opted for cannulation of the right femoral artery (FA) and the ascending aorta for CPB. A median sternotomy was performed, and intraoperative echocardiography confirmed the characteristics of the ascending aorta. However, due to extreme narrowing of the true lumen and significant mobility of the intimal flap, ascending aortic cannulation proved technically challenging. Also, regarding axillary artery cannulation, the true lumen was extremely narrowing due to dissection. Consequently, CPB was initiated via right FA arterial cannulation (18Fr PCKC-A, MERA, Tokyo, Japan) and bicaval venous cannulation (26Fr INKN-L, Medtronic, USA), employing the Heart Assist System III (HAS III, Mera Corporation, Tokyo, Japan). The extracorporeal circuit composition was that centrifugal pump (MERA Centrifugal Pump HCF-MP23, SENKO MEDICAL INSTRUMENT, Inc., Tokyo, Japan), FX-25 oxygenator (Terumo Corporation, Japan) were utilized. The arterial tubing size was 3/8 inch. A CDI Blood Parameter Monitoring System 500 (Terumo, Tokyo, Japan) was recalibrated every 60 min, and an arterial blood gas sample was also checked every 60 min. Alpha STAT was utilized. CPB was commenced with gradual flow increments. After achieving the total flow, we watched for 1 min to confirm no change parameters such as both mABP and TOI change rates. The patient was cooled to a target rectal temperature of 26 °C. During the early CPB phase, mABP and TOI change rates showed no significant alterations (Figs. 3 and 4). Cardiac index (CI) was maintained at 2.6–3.0 L/min/m^2^. The TOI and ΔO_2_Hb in the left lower limb gradually improved (Fig. 3b). However, approximately 15 min after CPB initiation, a significant bilateral disparity of 15 mmHg in radial arterial mABP was observed. Simultaneously, cerebral TOI and ΔO_2_Hb began to decline (Figs. 3 and 4). With rectal and tympanic temperatures around 32–34 °C, circulatory arrest was initiated at 30 °C. To mitigate malperfusion, we transitioned from NPF to PF. The pulse pressure of left radial mABP ranged from 20 to 25 mmHg (Fig. 5), with PF settings of 63 bpm heart rate, 100% base flow, and 50% duration. Arterial lactate levels remained stable at 1.1 mmol/L.

The transition to PF required approximately 3 min, during which the right radial mABP dropped to 29 mmHg before gradual recovery, accompanied by improvements in cerebral TOI (Figs. 3 and 4). Cerebral TOI nadirs were 85% (left) and 67% (right), with maximum declines in both cerebral TOI of −15%, ΔO_2_Hb of −6 μmol/L (right) and −8 μmol/L (left). Concurrently, deoxygenated hemoglobin (ΔHHb) levels showed a downward trend.

At a rectal temperature of 30 °C, circulatory arrest was established, PF ceased, and retrograde cerebral perfusion (RCP) initiated. During RCP, cerebral TOI and ΔO_2_Hb decreased, while ΔHHb levels rose. Upon initiating selective cerebral antegrade perfusion (SCP), cerebral TOI and ΔO_2_Hb improved, and ΔHHb levels normalized (Fig. 3a). Radial mABP discrepancies resolved during SCP (Fig. 4). SCP was maintained with a flow rate of 800–900 mL/min, radial mABP of 32–40 mmHg, and an LCCA perfusion pressure of 30 mmHg. The heat exchanger for the cerebral perfusion line was set at 20 °C. Cardiac arrest was achieved with retrograde cold blood cardioplegia by using glucose-insulin-potassium (GIK) solution (the ratio of blood 4: crystalloid 1), re-dosed (the ratio of blood 5: crystalloid 1) every 30 min.

The descending aorta was replaced with a 24 mm J-graft (Japan Lifeline or Triplex, Terumo Corporation, Tokyo, Japan). Circulation was restarted, and rewarming commenced. RCP, SCP, and circulatory arrest durations were 2, 21, and 27 min, respectively. Post-rewarming arterial lactate levels were 3.6 mmol/L. The Bentall procedure was completed using a 21 mm INSPIRIS RESILIA aortic valve (Edwards Lifesciences, Irvine, CA, USA). CPB was weaned uneventfully, with total CPB and aortic cross-clamp times of 198 and 161 min, respectively.

The patient’s postoperative course was uneventful. She was discharged on postoperative day 26 following a two-day intensive care unit stay.

Informed consent was obtained for the publication of along with the waived patient data and associated images.

## Discussion

TAAD outcomes are significantly influenced by the extent and location of the dissection, with reported mortality rates ranging from 15% to 30% [1]. When TAAD is complicated by malperfusion, the mortality rate increases to as high as 43%, with lower-extremity malperfusion occurring in approximately 20–30% of cases, and the preoperative presence of limb ischemia has been shown to double the risk of mortality and adversely impact long-term survival [2–6].

The selection of an appropriate cannulation strategy in TAAD necessitates meticulous planning, as certain techniques may exacerbate complications. Femoral artery cannulation, while advantageous due to its accessibility and familiarity to surgeons, poses risks such as malperfusion, retrograde perfusion-induced aortic branch vessel ischemia, propagation of the dissection flap, or atheroembolic events [7]. Alternatively, ascending aortic cannulation, including echocardiography-guided techniques or a combination of right axillary and femoral artery cannulation, has demonstrated improved safety profiles and favorable postoperative outcomes [8, 9].

In this complex case of TAAD with lower limb malperfusion, CPB was established via right femoral cannulation. During the initial phase of CPB, no significant differences were observed in mABP or TOI change rates. However, TOI and ΔO_2_Hb in the left lower limb gradually improved following CPB initiation. Approximately 15 min into CPB, 15 mmHg of a marked bilateral discrepancy in mABP between the radial arteries became evident, coinciding with declining cerebral TOI and ΔHbO_2_. To address malperfusion, we transitioned from NPF to PF, resulting in a gradual improvement in cerebral TOI.

The efficacy of PF in mitigating cerebral malperfusion in TAAD cases remains uncertain, with limited data available. Current CPB guidelines suggest that PF may reduce postoperative pulmonary and renal complications and recommend its use for patients at high risk of adverse outcomes (Class IIa, Level of Evidence B) [10–12]. Although its impact on cerebral outcomes is less established, studies such as O’Neil et al. have demonstrated that microcirculatory perfusion is better preserved with PF compared to NPF during CPB [13]. Nevertheless, the broader question of whether PF offers definitive advantages over NPF during CPB remains unresolved [14, 15].

One study indicated that patients with significant cerebrovascular stenotic lesions (≥75% stenosis or multiple prior cerebral infarctions) undergoing aortic surgery experienced fewer cerebrovascular accidents with PF compared to NPF over a 54-month follow-up period. In contrast, Murkin et al. reported no significant difference in cerebrovascular accident rates between PF and NPF in a similar cohort [16, 17]. İpek et al. observed no differences in neurocognitive outcomes but noted potential cerebral perfusion benefits of PF based on S100β protein levels and near-infrared spectroscopy (NIRS) values [18]. Reductions in regional cerebral oxygen saturation (rSO_2_) from baseline are associated with increased risks of postoperative cognitive dysfunction (POCD), delirium, and prolonged intensive care unit stays [19–21].

In our case, the lowest cerebral TOI values reached 85% (left) and 67% (right), with TOI reductions from the baseline of approximately 10%. Interventional thresholds for NIRS changes typically include 10–20% reductions from baseline or absolute values below 50% [22–24]. Following the switch to PF, cerebral TOI improved (left cerebral TOI: from 85 to 97%, Right cerebral TOI: from 67 to 77), and the decline in ΔO_2_Hb stabilized, likely due to enhanced oxygen transport and microcirculatory perfusion. The right mABP, which reached a nadir of 29 mmHg, also improved gradually until 42 mmHg. While studies have shown no significant differences in mABP between PF and NPF, indexed systemic vascular resistance (SVRi) during aortic cross-clamping was significantly lower with PF [14–18, 25].

Finally, the pulsatile method for upper body malperfusion remains a concern in its usefulness because NIRO improved, but it does not confirm improving true lumen blood flow by using echo. Therefore, this method is unprecedented, and it is uncertain whether similar results can be achieved. Also, this technique may not work in all instances, this case report is meant to remind others to keep in mind that PF is an option we possess as a potential solution in malperfusion cases.

## Data Availability

All available data are incorporated into the article.
